# Exploratory Retrospective Assessment of Patients with Psoriasis Receiving Biological Therapy

**DOI:** 10.3390/medicina62020257

**Published:** 2026-01-26

**Authors:** Andrada-Luciana Lazar, Sorana D. Bolboacă, Adrian-Lucian Baican, Corina-Iulia Baican, Sorina Dănescu, Elisabeta Candrea, Diana Valentina Câmpean, Paula Iluț, Ioana Semenescu, Adela-Viviana Sitar-Tăut, Romana Vulturar, Olga Hilda Orășan, Angela Cozma

**Affiliations:** 1 4th Medical Clinic, Internal Medicine Department, “Iuliu Haţieganu” University of Medicine and Pharmacy Cluj-Napoca, 400347 Cluj-Napoca, Cluj, Romania; andradalazarluciana@yahoo.com (A.-L.L.);; 2 Dermatology Department Integrated Ambulatory—Infectious Diseases Clinical Hospital Cluj-Napoca, 400001 Cluj-Napoca, Cluj, Romania; 3 Medical Informatics and Biostatistics, Department 11—Medical Education, Faculty of Medicine, “Iuliu Hațieganu” University of Medicine and Pharmacy Cluj-Napoca, 400349 Cluj-Napoca, Cluj, Romania; 4 Department of Dermatology, “Iuliu Hațieganu” University of Medicine and Pharmacy Cluj-Napoca, 400347 Cluj-Napoca, Cluj, Romaniasorinadanescu@yahoo.com (S.D.); elisabeta.candrea@umfcluj.ro (E.C.); 5 Department of Dermatology, County Emergency Hospital, 400535 Cluj-Napoca, Cluj, Romania; 6 Department of Cell and Molecular Biology, “Iuliu Haţieganu” University of Medicine and Pharmacy Cluj-Napoca, 400349 Cluj-Napoca, Cluj, Romania; romanavulturar@yahoo.co.uk

**Keywords:** psoriasis, Anti-Interleukin 17 biological therapy (anti-IL-17), Anti-Interleukin 23 biological therapy (anti-IL-23), Anti-Tumor Necrosis Factor biological therapy (anti-TNF), Psoriasis Area Severity Index (PASI), Dermatology Life Quality Index (DLQI)

## Abstract

*Background and Objectives*: Biological therapies improve disease severity and quality of life in patients with psoriasis, but data on Romanian patients remain limited. Our study aimed to characterize patients with psoriasis from Transylvania and to evaluate the impact of biologics on disease severity, treatment switching, affected special areas response, quality of life, and laboratory biomarkers. *Materials and Methods*: We conducted a retrospective exploratory study at two centers in Cluj-Napoca, Romania, using routinely collected medical data. *Results*: One-hundred and fifteen patients (aged 2–72 years) were evaluated; 45 patients received anti-TNF, 43 received anti-IL-17, and 27 received anti-IL-23. Patients treated with anti-IL-17 or anti-IL-23 were older at diagnosis than those treated with anti-TNF (*p* = 0.0001). Psoriatic lesions were prevalent in the scalp (58.3%) and nails (36.5%). Methotrexate was the most common prior systemic therapy (87.8%), with no difference between the groups (*p* = 0.7668). Patients receiving anti-TNF therapy (46.7%) or anti-IL-17 therapy (20.9%) also most frequently received prior treatment with systemic retinoids. Cardiometabolic comorbidities, including hypertension (40.9%) and diabetes mellitus (20.9%), were prevalent. Anti-IL-17 therapies were used more frequently in patients with hypertension (46.5%), diabetes mellitus (34.9%), and psoriatic arthritis (34.9%). Baseline severity scores were comparable across the groups (*p* > 0.10). A therapeutic switch occurred in approximately one-quarter of the patients, most frequently in the anti-TNF group (57.8%), which also showed higher PASI and DLQI scores at switching (*p* < 0.0001). At 36 weeks, anti-IL-17 and anti-IL-23 therapies demonstrated superior outcomes compared to anti-TNF therapy (*p* = 0.045). All patients receiving anti-IL-23 therapy achieved a PASI 100 at the 60-week follow-up. Significant improvements in PASI and DLQI were observed for all biologics (*p* < 0.0001). *Conclusions*: Biological therapies were associated with significant improvements in disease severity and quality of life. Anti-TNF therapies were switched more frequently due to reduced efficacy, while clinical improvement was observed regardless of lesion localization.

## 1. Introduction

Psoriasis is a chronic, immune-mediated inflammatory disease that primarily affects the skin and joints [1]. The prevalence of psoriasis in Europe, North America, and Africa is 4.6%, 3.7%, and 1.7%, respectively [2]. Differences also exist in psoriasis prevalence across Europe, with higher rates in high-income countries and in Central and Western Europe [3]. In Poland, psoriasis affects 1.7% of the population, with slightly higher rates in women than in men (1.8% in Polish women vs. 1.58% in Polish men) [4]. In Romania, the prevalence of psoriasis is 4.2–5.18% [5,6], while the highest reported rate is in Norway at 8.50% [7].

Plaque-type psoriasis is the most common form of the disease [8], whereas other variants include generalized pustular psoriasis (von Zumbusch type), localized pustular psoriasis of the palms and soles, acrodermatitis continua of Hallopeau, inverse psoriasis, guttate psoriasis, and erythrodermic psoriasis [8].

The diagnosis is clinical [9], and severity is assessed using the Psoriasis Area Severity Index (PASI) and Body Surface Area (BSA) [9]. Localizations of lesions are assessed using the Psoriasis Scalp Severity Index (PSSI) [10], Nail Psoriasis Severity Index (NAPSI) [11], and Genital Psoriasis Symptoms Scale (GPSS) [12]. Quality of life is evaluated using the Dermatology Life Quality Index (DLQI) [13]. The disease is considered severe when psoriatic lesions are localized in specific areas or when PASI and DLQI exceed 10 points [14].

Psoriasis etiopathogenesis involves an interaction between genetic predisposition and environmental triggers [15], affecting not only the skin but also the joints, cardiovascular and metabolic systems, and psychological well-being [1,16]. Over 109 susceptibility loci have been identified [17], and triggers include infections, trauma, drugs, and autoantigens [18]. Immune dysregulation in psoriasis involves both the innate and adaptive immunity [19]. The key pathway that perpetuates inflammation is the TNFα-IL-23-Th17 axis [20]. Interleukins that play a significant role in pathogenesis are targets of biological therapies [21].

Management includes topical treatment, phototherapy, systemic conventional agents, and biologics [14]. Biological therapy targets TNFα (Tumor Necrosis Factor-alpha), IL-17 (IL-interleukin), IL-23, or IL-12/23, which are key interleukins implicated in etiopathogenesis [14]. Treatment choices depend on severity, site involvement, and comorbidities, and must address both skin and joint disease, as well as associated conditions [14,22,23].

Patients with psoriasis have an increased risk of atherosclerotic disease compared to the general population [24], with higher intima-media thickness (IMT) and more frequent atherosclerosis than controls [25]. Early onset and severe psoriasis further increase cardiovascular risk [26], likely via the IL-23–IL-17 axis [27].

Few original studies have been conducted on patients with psoriasis in Romania. Dascălu et al. found that metabolic syndrome (MetS) is more common in patients with psoriasis [28]. Additionally, patients with psoriasis had a higher IMT and more frequent atherosclerotic plaques [28]. The percentage of carotid artery plaques is higher in patients with psoriasis and MetS [28]. Cardiovascular disease is the most frequent comorbidity, followed by hypertension, dyslipidemia, and diabetes mellitus [5,6]. Regarding treatment response, Bucur et al. reported greater early PASI improvement with Ixekizumab than Secukinumab [29]. Psoriasis also carries a substantial psychological burden [30,31], including depression, low self-esteem, anger [30], anxiety [31], stigma [6], and a reduced quality of life [6,32].

Studies on patients with psoriasis in Romania have focused on the prevalence, characteristics, and quality of life [4,5] without evaluation of evolution under biological therapies. In the context of this gap, our retrospective study had twofold aims: to evaluate the therapy-switching rate and evolution of biologic-naïve patients under biological therapy using severity scores over time, and to analyze lesions from special areas response and the effects, if any, of biological treatment on measured laboratory biomarkers and of the skin condition on the patient’s life reflected by the DLQI score. The primary endpoints were the proportion of biological-naïve patients with psoriasis who required a switch in biological therapy during follow-up and treatment effectiveness, assessed by PASI response (PASI 100, PASI ≥ 90, PASI ≥ 75, PASI ≥ 50, and PASI < 50) and changes in DLQI scores evaluated at 36 and 60 weeks of treatment. Secondary endpoints included time to clinical response of psoriatic lesions located in special areas and changes in laboratory parameters (glycemia, creatinine, serum urea, and serum lipid levels) from baseline to 36 and 60 weeks of follow-up.

## 2. Materials and Methods

The study has been approved by the following Ethics Committees: Infectious Diseases Clinical Hospital, Cluj-Napoca Ethics Committee, approval 20948/29.11.2024; “Iuliu Hațieganu” University of Medicine and Pharmacy, Cluj-Napoca Ethics Committee, approval 55460/5.07.2023; County Emergency Hospital, Cluj-Napoca Ethics Committee, approval 55460/27.11.2023. This study was conducted in accordance with the principles of the Declaration of Helsinki.

### 2.1. Study Design and Variables

This retrospective study was conducted at the Dermatology Department of the County Emergency Hospital, Cluj-Napoca, and the Integrated Ambulatory-Infectious Diseases Clinical Hospital, Cluj-Napoca. Biologically naïve patients with histopathologically proven psoriasis vulgaris who received biological treatment with anti-TNF, anti-IL-17, or anti-IL-23, between 2010 and 2025, were eligible for the study.

Data were retrospectively collected from hospital and electronic medical records, laboratory databases, and the Romanian National Registry for Dermato-Venereological Disorders between January 2025 and October 2025. In Romania, treatment decisions are individualized and guided by the national protocol, the “Therapeutic protocol for severe chronic psoriasis (plaque psoriasis)—biological agents and small molecule therapies with intracellular action” [33]. The choice of biological agent is made in compliance with current legislation, based on the clinical characteristics of the disease, the patient’s age, pre-existing comorbidities, and the treating dermatologist’s experience and local facilities’ capabilities [33]. For the initiation of biological treatment, the histopathological certification of psoriasis is mandatory [33]. Additionally, for the initiation of treatment and monitoring of patients undergoing biological therapy, several laboratory investigations are necessary (i.e., complete blood count, erythrocyte sedimentation rate, liver and kidney function tests, electrolytes (sodium, potassium), tuberculin skin test/Interferon-Gamma Release Assay (except for apremilast), lung X-ray, screening for hepatitis B and C) [33]. The patients must also present a certificate from their family doctor stating the chronic diseases they have [33]. According to the national protocol, psoriasis vulgaris is classified as severe when PASI > 10 or lesions are located in topographic regions associated with significant functional impairment and/or high level of suffering and/or difficult to treat: face, scalp, palms, soles, nails, genital region, large folds, quantified by area scores [33]. The inclusion criteria for the initiation of biological treatment for adult patients with psoriasis vulgaris are: the presence of severe plaque psoriasis defined as (involvement of more than 10% of the body or PASI ≥ 10 or lesions located in topographic regions associated with significant functional impairment and/or high level of suffering and/or difficult to treat: face, scalp, palms, soles, nails, genital region, large folds) for more than 6 months, together with a DLQI ≥ 10, eligibility for biological therapy and failure/intolerance/contraindication for conventional systemic therapy [33]. The therapeutic target is defined as a decrease in PASI score of 50% from baseline (including 50% of the specific scores for the special areas affected) with a long-term objective of reaching an average remission of lesions of 90% [33]. Regarding DLQI, a minimum 5-point decrease from baseline is expected, with a long-term objective of reaching an absolute value of no more than 2 [33]. Discontinuation of biological treatment is indicated when the therapeutic target has not been achieved, as assessed by the therapeutic target [33]. Discontinuation of treatment is also indicated in the event of a severe adverse reaction [33]. Biological treatment switch is required in the case of patients who, upon evaluation, do not reach or maintain the therapeutic target during treatment or who have developed an adverse reaction [33]. The biological agent can be changed to another biological agent from the same therapeutic class only once in succession [33]. The national protocol also includes recommendations for the pediatric population (age 4 to 18) for biological therapy for psoriasis vulgaris [33].

The extracted variables included demographic data (sex, age, ethnicity), clinical data (body mass index [BMI], presence of overweight or obesity, age at diagnosis, comorbidities at biological treatment initiation, treatment details, severity scores (PASI), and effect of skin condition on the patient’s life reflected by DLQI score), and routinely measured laboratory biomarkers (at the time of biological treatment initiation, at 36 weeks, and at 60 weeks). The raw data for laboratory measurements were collected as continuous variables and then transformed into a nominal variable, with values classified as normal, low, or high based on the normal values of the two laboratories, because different devices, laboratory kits, and ranges were used.

All data were anonymized before statistical analysis to maintain patient confidentiality.

### 2.2. Analysis of Data

An exploratory statistical analysis was conducted using the Jamovi (v. 2.6.26.0) software at a level of significance of 5%. Qualitative data are reported as numbers (percentages), and differences between groups (biological therapy) were tested using Fisher’s exact test. Distribution was tested using the Shapiro–Wilk test prior to analysis for all quantitative variables, and data were reported following a free-distribution analysis when the normality assumption was not met.

The PASI and DLQI scores were evaluated at different time points using Friedman’s test, followed by post hoc analysis using Dwass–Steel–Critchlow–Fligner for pairwise comparisons when Friedman’s test was statistically significant. The psoriasis area and severity index (PASI) score (range, 0–72) was used to classify disease severity as mild (0 < PASI ≤ 5), moderate (5 < PASI ≤ 10), or severe (PASI ≥ 11) at baseline. The effect of the skin condition on the patient’s life was reported to have no effect (0 < DLQI ≤ 1), small effect (2 < DLQI ≤ 5), moderate effect (6 < DLQI ≤ 10), very large effect (11 < DLQI ≤ 20), and extremely large effect (21 < DLQI ≤ 30). Correlation analysis between baseline BMI, baseline PASI, and DLQI scores was performed using Spearman’s correlation coefficient. Graphical representations were constructed using Microsoft Excel.

No imputation method was used to handle the missing data, and the number of patients per group was reported whenever missing data were present.

## 3. Results

**Figure 1:**
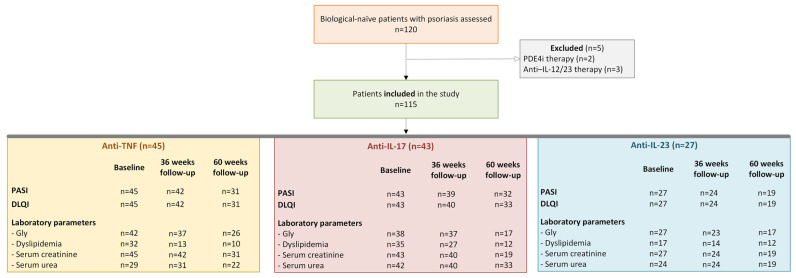
Flow diagram of patient selection and follow-up. n- the number of patients; Gly—glycemia; PASI—Psoriasis Area Severity Index; DLQI—The Dermatology Life Quality Index; Anti-TNF—anti-tumor necrosis factor alpha biological therapy; Anti-IL-17—anti-interleukin 17 biological therapy; Anti-IL-23—anti-interleukin 23 biological therapy.

One hundred and twenty biologic-naïve patients with a histopathological diagnosis of psoriasis and eligible for biological therapy were assessed for eligibility. Of these, 115 patients aged 2–72 years were included in the study. Five patients were excluded: two because of prior PDE4 inhibitor therapy and three because of prior anti-IL-12/23 biological treatment. The included patients were allocated to the following biological treatment groups: anti-TNF (n = 45), anti-IL-17 (n = 43), and anti-IL-23 (n = 27). Fifty-four patients received biological therapy within 6 months of disease onset, with a time span of 1–38 years from disease onset to biological therapy initiation. The flow diagram of patient selection and follow-up is presented in Figure 1.

### 3.1. Characteristics of the Evaluated Cohort

The characteristics of the patients undergoing biological treatment are presented in Table 1. The most prevalent comorbidity was hypertension, followed by diabetes mellitus (Table 1), with a heterogeneous distribution according to biological therapy class. A limited number of patients had heart failure (two patients), ischemic cardiomyopathy (five patients), myocardial infarction (one patient), stroke (one patient), peripheral artery disease (one patient), neoplasia (five patients), chronic kidney disease (one patient), tuberculosis (six patients), or infection (five patients). All patients received topical treatment before biologics, except for three; the rest received systemic conventional therapy.

Baseline BMI data was available for 88 patients. No statistically significant associations were found between baseline BMI and baseline PASI (Spearman’s ρ = −0.14, *p* = 0.2053) or between baseline BMI and baseline DLQI (Spearman’s ρ = −0.05, *p* = 0.6422)

**Figure 2:**
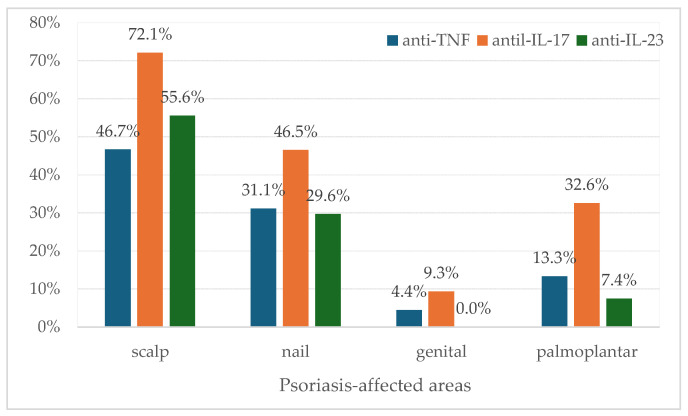
Distribution of psoriasis-affected regions before initiation of biological therapy. Statistical significance between groups was observed only for palmoplantar (Fisher’s exact test: *p* = 0.0191) and for scalp (Fisher’s exact test: *p* = 0.0501).

The most prevalent psoriatic lesions were localized to the scalp (58.3%) and nails (36.5%) (Figure 2).

### 3.2. Disease Severity Dynamics Under Biological Treatment

A biological switch was observed in 29 patients: 26 (57.8%) had initial anti-TNF (17 cases with one switch and nine cases with two switches) and three (7.0%, with a single switch per case) anti-IL-17 (Fisher’s exact test: *p* < 0.0001). The switch was due to ineffectiveness (26 cases), multiple sclerosis (one case, under anti-TNF therapy), and paradoxical psoriasis (another case, under anti-TNF therapy). The need for switching occurred between 1 and 12 months after initiation, with a median of 6 months and an interquartile range (IQR) of 5 to 10 months.

Similar baseline PASI scores were observed across the biological treatment groups, with higher values at the time of therapy switch and at 36 and 60 weeks of follow-up for patients with anti-TNF (Table 2). A change towards mild PASI scores and no effects on quality of life were observed in all groups (Table 2).

**Figure 3:**
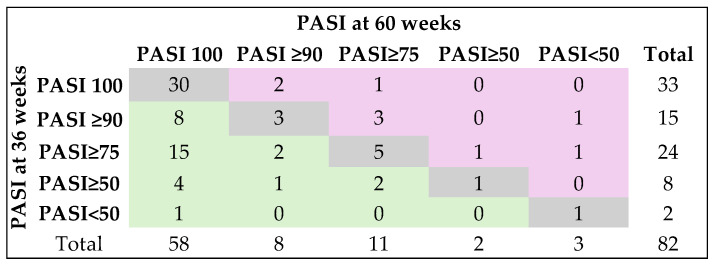
Changes in PASI between weeks 36 and 60 in the evaluated cohort. Gray color (diagonal cells) indicates no change in response category (patients stayed in the same PASI response group at week 60 as they were at week; Green color (cells to the left of the diagonal) indicates improvement (patients moved to a better PASI category by week 60 of follow-up; Pink color (cells to the right of the diagonal) indicates worsening (patients moved to a lower PASI category by week 60 compared with week 36 of follow-up).

Regardless of the biological treatment group, the PASI score changed significantly from baseline to 60 weeks follow-up, with statistically significant differences between pairs of determinations (*p* < 0.0001), with one exception observed in the anti-IL-17 group, where PASI scores at 36 weeks were not statistically significant compared to those at 60 weeks follow-up (Durbin-Conover Pairwise comparisons: *p* = 0.1295). PASI remained the same at 60 weeks compared to 36 weeks for 40 (48.8%) patients, changed positively in 33 (40.2%) patients, and negatively in nine (11%) patients (analysis conducted on 82 patients) (Fisher’s exact test: *p* = 0.0201) (Figure 3).

**Figure 4:**
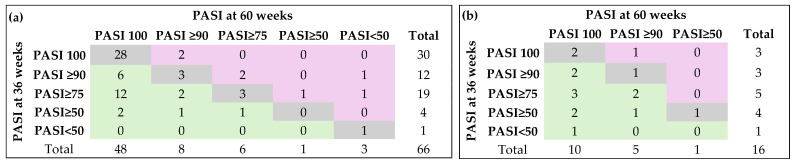
Dynamics of PASI score: (**a**) patients without biological therapy switching (n = 66); (**b**) patients with biological therapy switching (n = 16). Gray color (diagonal cells) indicates no change in response category (patients stayed in the same PASI response group at week 60 as they were at week; Green color (cells to the left of the diagonal) indicates improvement (patients moved to a better PASI category by week 60 of follow-up; Pink color (cells to the right of the diagonal) indicates worsening (patients moved to a lower PASI category by week 60 compared with week 36 of follow-up).

The change in PASI score was statistically significant only in patients who did not switch to therapy (Figure 4a, Fisher’s exact test: *p* = 0.0046).

The PASI scores decreased over time, but the values remained higher in patients with lesions in specific locations than in those without lesions (Table 3).

Adverse events were observed in two patients receiving topical therapy, in three patients who developed pruritus and erythema after phototherapy, and in 24 patients receiving systemic therapy. Treatment with MTX was associated with cytopenia (one case), digestive intolerance (three cases), hepatic cytolysis (eight cases), nausea (two cases), cholestasis (one case), and pulmonary fibrosis (two cases). Treatment with cyclosporine was associated with increased creatinine (one case), hypertension (four cases), and osteodynia (one case). Retinoid treatment was associated with metabolic syndrome (three cases), osteodynia (one case), abdominal pain (two cases), digestive adverse events (one case), and hepatic cytolysis (one case).

### 3.3. Special Areas Response, Patient-Reported Outcomes, and Biomarker Modulation

**Figure 5:**
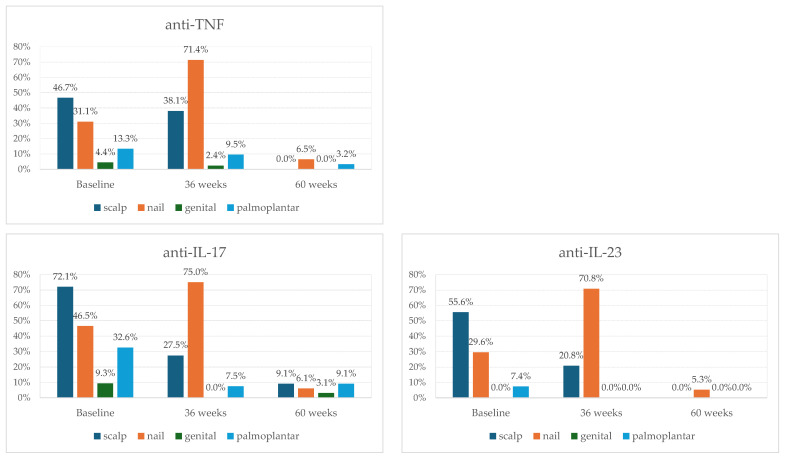
Dynamics of psoriasis-affected special areas by class of the initial biological therapy (n = 115 baseline, n = 106 at 36 weeks of follow-up, and n = 83 at 60 weeks of follow-up).

Biological therapy improved disease manifestations, independent of the initial biological agent used (Figure 5).

Fridman’s ANOVA identified significant changes in the DLQI scores at baseline, at the switch, and at 60 weeks of follow-up (Table 4). A change towards no effects on quality of life at 36 and 60 weeks of follow-up was observed in all groups (Table 4).

The DLQI scores decreased over time, regardless of lesion localization, with higher scores observed in patients with lesions localized to special areas than in those without lesions (Table 5).

Routinely assessed biomarkers showed a similar proportion of elevated values across all initial biological therapy classes, except for glucose metabolism at baseline (see GLY-serum glucose level, Table 6).

## 4. Discussion

The evaluated biological therapies were associated with significant improvements in disease severity and quality of life. Anti-TNF therapies were switched more frequently due to reduced efficacy, while clinical improvement was observed regardless of lesion localization.

### 4.1. Findings Interpretation

Patients treated with anti-IL-17 and anti-IL-23 in our cohort were older at diagnosis than those treated with anti-TNF (Table 1). Hypertension was the most common comorbidity among the patients with psoriasis, followed by diabetes mellitus (Table 1). Most of the patients with psoriatic arthritis received anti-IL-17 therapy (Table 1). Psoriasis is a chronic inflammatory disease that affects the body beyond the skin [34], and its management is multidisciplinary, with attention to comorbidities [35]. Biological therapies targeting key interleukins influence comorbidities and mortality risk, and improve quality of life and psychological burden [36,37,38]. Genetic and environmental factors can trigger or worsen the disease [15].

In our cohort, BMI was not significantly associated with baseline PASI or DLQI scores, in contrast to other studies that reported that higher BMI correlates with greater disease severity and lower quality-of-life scores [39,40]. Wang et al. [39] reported in a multicenter study of 1979 patients that each 1-point increase in BMI is associated with higher DLQI, PASI, and BSA scores [39]. Obesity and overweight are linked to increased disease severity, as reflected by higher PASI scores [40] and diminished efficacy of biological therapies [41]. Obesity is recognized as both a cause and a modifier of psoriatic diseases [42]. Baseline BMI did not show a significant association with PASI or DLQI in our cohort, potentially due to variable overweight and obesity prevalence and missing data, considering that we had data only for 61.7% of the evaluated cohort.

Cardiometabolic disorders were the most common comorbidities in the evaluated cohort (Table 1), consistent with a study conducted by Almenara-Blasco et al. [43]. Hypertension was the most common comorbidity observed in a Romanian study by Nicolescu et al. [5], in which infections ranked second, and dyslipidemia third [5]. Diabetes mellitus was the second most prevalent comorbidity in our cohort, compared to the third position reported by Boca et al. [6].

Topical therapy, which is considered safe [44], caused reactions in only two patients in our cohort. Phototherapy led to three acute events (pruritus and erythema). Conventional systemic therapy was associated with the highest number of adverse reactions. In the case of methotrexate, which was prescribed to the majority of patients, the most frequent adverse reactions were digestive, and hepatic cytolysis syndrome was the most frequent. West et al. ranked infections as the most common adverse reaction in patients treated with methotrexate in a meta-analysis [45]; this was not described in the cohort of this study.

Severity scores were comparable across treatment groups at baseline, while at the therapeutic switch, the anti-TNF group had higher PASI and DLQI scores than the anti-IL-17 group (Table 2 and Table 4). No therapeutic switch was required for patients treated with anti-IL-23 biologics. In our study, 29 treatment switches were recorded: 26 with anti-TNF and three with anti-IL-17 therapies, mainly due to loss or lack of efficacy. Anti-TNF-α was also the most frequently switched class in real-world data from Armstrong et al. [46]. The mean time to the first switch was 6 months, similar to the 6.5 months reported by Mease et al. [47], with inefficacy as the leading cause [47].

At 36 weeks, the anti-IL-17 and anti-IL-23 classes were associated with greater benefits than anti-TNF (Table 2). All patients who received anti-IL-23 therapy reached PASI 100 at 60 weeks of follow-up and PASI ≥ 50 at 36 weeks of follow-up (Table 2). In contrast, two patients with anti-IL-17 had a PASI < 50 at 60 weeks of follow-up. In the anti-TNF-α group, only 21.4% reached PASI 100 at 36 weeks, compared to 43.6% for anti-IL-17 and 54.2% for anti-IL-23. Two anti-TNF patients showed increased PASI scores at 36 weeks (Table 2, Figure 2 and Figure 3). The literature demonstrates that anti-IL-17 and anti-IL-23 biologics most effectively reduce PASI in biologically naïve patients at 3, 6, and 12 months [48], with Ixekizumab and Secukinumab providing superior 24-week responses [49]. Ntawuyamara et al. [49] reported no statistically significant differences in DLQI scores among biologics. In our study, the DLQI showed the best results at 36 and 60 weeks of follow-up in patients receiving anti-IL-23 biologics (Table 4). Despite the favorable therapeutic outcomes observed in the anti-IL-23 group, consistent with the literature, this group included the smallest number of patients compared with the other two groups.

Our results indicated that scalp lesions responded more favorably to anti-TNF and anti-IL-23 therapies (Table 3 and Table 5, Figure 5). It is well known that the most significant impact of biological therapies is on lesion localization, given their effects on functionality and quality of life [50]. Identifying a therapy that leads to the fastest and sustained improvement is essential [50]. Khan et al. [51] reported the greatest mean NAPSI improvement at week 24 with Brodalumab, followed by Etanercept and Ixekizumab [51]. The highest probability of complete nail clearance was achieved with Adalimumab (44%), followed by Ixekizumab (41%), and Brodalumab (31.6%) [51]. At the 52-week evaluation, the greatest mean NAPSI improvement was observed in patients who received Ixekizumab (83.3% of patients), followed by Brodalumab (83.1% of patients), and Adalimumab (70.2% of patients) [51]. The highest proportion of patients with NAPSI 0 was for those who received Brodalumab, followed by Adalimumab (58.8%), Ixekizumab (57.5%), and Ustekinumab (33.9%) [51]. For scalp psoriasis, PSSI 100 was achieved by week 16 with Ixekizumab (74.6% of patients) and Secukinumab (35% of patients), while PSSI75 was achieved at week 8 in >75% of patients on Ixekizumab and Infliximab [52]. Ustekinumab and Risankizumab led to 94% and 90% reductions in PSSI at the 12-week evaluation, and Adalimumab reduced PSSI by 77.2% after 16 weeks of treatment [52]. In our cohort, nail improvement was the greatest in patients who were treated with anti-IL-23 and anti-IL-17 (Figure 5).

Regarding the laboratory parameters, at baseline, most patients with higher glycemia received anti-IL-17 and anti-IL-23 therapies (Table 6) without any other impact on the evaluated biomarkers, regardless of the biologicals. Guselkumab is known to lower non-HDL cholesterol, whereas Secukinumab and Adalimumab affect glucose levels [49]. In our study, biologics did not significantly affect the lipid profile, glycemia, or renal function (Table 6), but the generalizability of the findings is limited due to the existence of missing data.

### 4.2. Strengths and Limitations of the Study

One of the strengths of this study is that the patients were recruited from two medical centers, reflecting prescribing patterns across multiple physicians regarding patients’ comorbidities at the initiation of biological therapy. The cohort included patients from both urban and rural areas in Central and Northern Romania, thus enhancing the representativeness of the results for the psoriasis population in Transylvania. Additionally, this study provides insight into real-world prescription patterns of biological therapies, an area with limited published data, particularly in Eastern Europe and Romania.

Although this study provides valuable insights, certain limitations must be acknowledged. The study design, based on routinely collected medical data, is limited by available data and associated quality, leading to potential biases, such as selection bias, measurement or instrument, misclassification, and admission rate bias. Consequently, missing and incomplete data reduced the statistical power of our study and the generalizability of the findings outside the evaluated cohort. Moreover, in the absence of control for confounding factors, considering that certain clinically relevant variables are not routinely documented, an adjusted analysis was not conducted. The small number of patients allowed only analysis by therapy class and not by individual drugs. Given the retrospective nature of the study and the fact that the subjects were selected from two medical centers, the exact timing of patient assessment may vary; therefore, patients may not be assessed exactly at 36 and 60 weeks of follow-up. Since biomarkers were evaluated in different laboratories, we reported nominal values rather than measured values, which decreased the power of the reported results. In addition, lesion localization was recorded only as present and not according to severity. Finally, Romanian national legislation and guidelines for biological treatments for psoriasis changed over time, which also affected access to biologics and the use of specific scoring systems for lesion localization [33,53,54,55,56]. Potential selection bias, center-specific factors, and attrition during follow-up may affect the generalizability of our findings.

## 5. Conclusions

In the evaluated cohort, patients with psoriasis who received biological therapy showed a favorable clinical course. The limited number of patients receiving anti-IL-23 therapy achieved the highest PASI 100 rates, with all patients reaching this by week 60 of follow-up. Anti-TNF was the most frequently switched treatment, primarily due to loss of efficacy, whereas no anti-IL-23 patients required a switch. Biological therapy had no significant impact on the lipid profile, blood glucose, or renal function.

## Figures and Tables

**Table 1:** Baseline patient characteristics by initial biological treatment class.

Variable	Anti-TNF (n = 45)	Anti-IL-17 (n = 43)	Anti-IL-23 (n = 27)	Stat. (*p*-Value)
Age at diagnosis, years	22 [14 to 33]	43.5 [27.3 to 53.5], n = 42	35 [19 to 45], n = 25	17.9 (0.0001) *
Living place				n.a. (0.5246)
Rural	14 (31.1)	11 (25.6)	5 (18.5)	
Urban	31 (68.9)	32 (74.4)	22 (81.5)	
Ethnicity	n = 44			n.a. (0.8515)
Romanian	38 (86.4)	36 (83.7)	22 (81.5)	
Hungarian	6 (13.6)	7 (16.3)	5 (18.5)	
Employment status	n = 42	n = 40		n.a. (0.4007)
Employed	28 (66.7)	24 (60)	14 (51.9)	
Retired	12 (28.6)	16 (40)	12 (44.4)	
Student	2 (4.8)	0 (0)	1 (3.7)	
Behavior				
Smoking	2 (4.4)	6 (14)	3 (11.1)	n.a. (0.3056)
Alcohol consumption	0 (0)	2 (4.7)	1 (3.7)	n.a. (0.3608)
Body mass index class	n = 33	n = 22	n = 16	
Overweight	16 (35.6)	8 (18.6)	7 (25.9)	n.a. (0.2149)
Obesity	17 (37.8)	14 (32.6)	9 (33.3)	n.a. (0.8785)
Comorbidities				
Hypertension	12 (26.7)	20 (46.5)	15 (55.6)	6.7 (0.0345)
Diabetes mellitus	7 (15.6)	15 (34.9)	2 (7.4)	n.a. (0.0140)
Dyslipidemia	3 (6.7)	7 (16.3)	2 (7.4)	n.a. (0.3629)
Histology				
Acanthosis	44 (97.8)	39 (90.7)	27 (100)	n.a. (0.2057)
Elongated epidermal rete ridges	40 (88.9)	25 (58.1)	25 (92.6)	n.a. (0.0003)
Hypogranulosis	42 (93.3)	33 (76.7)	25 (92.6)	n.a. (0.0561)
Munro microabscess	33 (73.3)	20 (46.5)	18 (66.7)	n.a. (0.0302)
Ortokeratosis	0 (0)	4 (9.3)	0 (0)	n.a. (n.a.)
Parakeratosis	43 (95.6)	40 (93)	26 (96.3)	n.a. (0.8770)
Hyperkeratosis	41 (91.1)	32 (74.4)	25 (92.6)	n.a. (0.0543)
Inflammatory infiltrate				n.a. (0.0004)
mild	11 (24.4)	26 (60.5)	9 (33.3)	
moderate	34 (75.6)	14 (32.6)	18 (66.7)	
pronounced	0 (0)	1 (2.3)	0 (0)	
Psoriatic arthritis	5 (11.4), n = 44	15 (34.9)	6 (23.1)	n.a. (0.0139)
… before biologics				
Topical corticosteroids	45 (100)	39 (90.7)	25 (92.6)	n.a. (0.1109)
Topical Vitamin D analogs	37 (82.2)	36 (83.7)	23 (85.2)	n.a. (>0.9999)
Emollients	44 (97.8)	41 (95.3)	26 (96.3)	n.a. (0.8337)
Phototherapy	22 (48.9)	29 (69.0)	20 (74.1)	n.a. (0.0646)
MTX	39 (86.7)	37 (86.0)	25 (92.6)	n.a. (0.7668)
Cyclosporine	5 (11.1)	10 (23.3)	4 (14.8)	n.a. (0.3120)
Systemic retinoids	21 (46.7)	9 (20.9)	0 (0)	n.a. (<0.0001)

Data are reported as numbers (%), except for age at diagnosis, when the value of median and [Q1 to Q3] were reported (where Q1 is the 25th percentile and Q3 is the 75th percentile). The number of patients evaluated (n) was reported when missing data were present. n.a. indicates application of Fisher’s exact test. Post hoc Dwass–Steel–Critchlow–Fligner pairwise comparisons: * *p* < 0.0001 for anti-TNF vs. anti-IL-17; MTX: methotrexate.

**Table 2:** Severity scores by type of initial biological treatment.

Variable	Anti-TNF (n = 45)	Anti-IL-17 (n = 43)	Anti-IL-23 (n = 27)	Stat. (*p*-Value)
PASI score				
Baseline	21.5 [18.5 to 26.8]	19.5 [15.2 to 30.3]	19.3 [16.5 to 21.3]	3.1 (0.2145)
At switch	10.1 [3 to 15.7], n = 44	3 [3 to 3]	3 [3 to 3]	42.8 (<0.0001) ^#^
PASI at 36 weeks follow-up				n.a. (0.0450) *
PASI 100	9 (21.4)	17 (43.6)	13 (54.2)	
PASI ≥ 90	5 (11.9)	11 (28.2)	3 (12.5)	
PASI ≥ 75	14 (33.3)	7 (17.9)	5 (20.8)	
PASI ≥ 50	9 (21.4)	3 (7.7)	3 (12.5)	
PASI < 50	3 (7.1)	1 (2.6)	0 (0)	
INCREASE	2 (4.8)	0 (0)	0 (0)	
PASI at 60 weeks follow-up				n.a. (0.0103)
PASI 100	21 (67.7)	18 (56.3)	19 (100)	
PASI ≥ 90	1 (3.2)	7 (21.9)	0 (0)	
PASI ≥ 75	7 (22.6)	4 (12.5)	0 (0)	
PASI ≥ 50	1 (3.2)	1 (3.1)	0 (0)	
PASI < 50	1 (3.2)	2 (6.3)	0 (0)	

PASI scores are reported as median [Q1 to Q3], where Qs represents the values of the quartiles, all other variables are reported as numbers (%), and *n* is the number of patients with raw data. n.a.—not applicable, * Fisher’s exact test and Monte Carlo simulation. ^#^ Post hoc Dwass–Steel–Critchlow–Fligner pairwise comparisons: *p* < 0.0001 for anti-TNF vs. anti-IL-17, and anti-TNF vs. anti-IL-23.

**Table 3:** Dynamics of PASI scores by lesion localization.

Lesion Localization	Baseline	Follow-Up	
		36 Weeks	60 Weeks
Scalp			
Absent	21.4 [18 to 26.8]	0 [0 to 2.4]	0 [0 to 0.7]
Present	19.5 [15.5 to 27.4]	4.5 [2.2 to 6.1]	2.7 [1.5 to 19.5]
Stat. (*p*-value)	1359 (0.1578)	491 (<0.0001)	35 (0.0111)
Nail			
Absent	20.3 [17.3 to 26.2]	0.7 [0 to 3.5]	0 [0 to 0.6]
Present	20.5 [12.6 to 29]	3.8 [1.2 to 6]	4.5 [0.3 to 9.6]
Stat. (*p*-value)	1461 (0.6758)	688 (0.0019)	78 (0.0058)
Genital			
Absent	20.2 [16.4 to 26.3]	1.6 [0 to 5]	0 [0 to 0.6]
Present	28.8 [22.5 to 34.2]	20.4 [20.4 to 20.4]	36.2 [36.2 to 36.2]
Stat. (*p*-value)	201 (0.113)	2 (0.0936)	0 (0.0355)
Palmoplantar			
Absent	20.3 [17 to 26.3]	1.2 [0 to 4.6]	0 [0 to 0.5]
Present	20.2 [13.9 to 31.6]	6 [3.7 to 10.8]	6.3 [2.8 to 16.3]
Stat. (*p*-value)	1022 (0.9943)	123 (0.0036)	26 (0.0005)

**Table 4:** Dynamics of DLQI scores by type of initial biological treatment.

Variable	Anti-TNF (n = 45)	Anti-IL-17 (n = 43)	Anti-IL-23 (n = 27)	Stat. (*p*-Value)
DLQI				
Baseline	22 [19 to 24]	17 [13 to 20]	21 [13.5 to 22.5]	13.2 (0.0014) *
At switch	11.2 [3 to 20], n = 44	3 [3 to 3]	3 [3 to 3]	34.9 (<0.0001) ^#^
36 weeks follow-up	2 [0 to 4], n = 42	1 [0 to 3], n = 40	0 [0 to 2], n = 24	5.8 (0.054)
60 weeks follow-up	0 [0 to 1], n = 31	0 [0 to 2], n = 33	0 [0 to 0], n = 19	11.3 (0.0036) ^^^
DLQI baseline				n.a. (0.0022)
moderate effect	18 (40)	32 (74.4)	13 (48.1)	
extremely large effect	27 (60)	10 (23.3)	14 (51.9)	
DLQI at switch	n = 44	n = 43	n = 27	n.a. (<0.0001)
small effect	20 (45.5)	40 (93)	27 (100)	
moderate effect	14 (31.8)	3 (7)	0 (0)	
extremely large effect	10 (22.7)	0 (0)	0 (0)	
DLQI at 36 weeks follow-up	n = 42	n = 40	n = 24	n.a. (0.1686)
no effect	14 (33.3)	23 (57.5)	14 (58.3)	
small effect	21 (50)	12 (30)	9 (37.5)	
moderate effect	6 (14.3)	5 (12.5)	1 (4.2)	
extremely large effect	1 (2.4)	0 (0)	0 (0)	
DLQI at 60 weeks follow-up	n = 31	n = 33	n = 19	n.a. (0.0299)
no effect	24 (77.4)	23 (69.7)	19 (100)	
moderate effect	7 (22.6)	9 (27.3)	0 (0)	
extremely large effect	0 (0)	1 (3)	0 (0)	

DLQI scores are reported as median [Q1 to Q3], where Qs represents the values of the quartiles, and all other variables are reported as numbers (%). The number (*n*) of patients with raw data is reported when missing data were present. Post hoc Dwass–Steel–Critchlow–Fligner pairwise comparisons: * *p* = 0.0011 for anti-TNF vs. anti-IL-17; ^#^ *p* < 0.0001 for anti-TNF vs. anti-IL-17 and anti-TNF vs. anti-IL-23; ^^^ *p* = 0.018 for anti-TNF vs. anti-IL-23, and *p* = 0.002 for anti-IL-17 vs. anti-IL-23; n.a. not applicable, comparison between groups in case of qualitative data was mane with Fisher’s exact test.

**Table 5:** Dynamics of DLQI scores by lesion localization.

Lesion Localization	Baseline	Follow-Up	
		36 Weeks	60 Weeks
Scalp			
Absent	21 [15 to 25]	0 [0 to 2]	0 [0 to 1]
Present	19 [13.5 to 22]	3 [2 to 4]	2 [1 to 3.5]
Stat. (*p*-value)	1288 (0.0694)	562 (<0.0001)	65 (0.0978)
Nail			
Absent	20 [15 to 23]	0 [0 to 3]	0 [0 to 1]
Present	19 [13 to 22.8]	2 [1 to 4]	2 [0 to 4]
Stat. (*p*-value)	1363 (0.3239)	761 (0.0085)	115 (0.0589)
Genital			
Absent	20 [14 to 23]	2 [0 to 3]	0 [0 to 1]
Present	19.5 [15.3 to 21.5]	18 [18 to 18]	5 [5 to 5]
Stat. (*p*-value)	316 (0.8947)	2 (0.0876)	2 (0.0428)
Palmoplantar			
Absent	20 [15 to 24]	2 [0 to 3]	0 [0 to 1]
Present	17 [12 to 19.8]	3 [1 to 4.5]	4 [3.3 to 4.3]
Stat. (*p*-value)	601 (0.0027)	263 (0.2669)	23 (0.0004)

**Table 6:** Distribution of patients with elevated biomarker levels by initial biological therapy class.

Variable	Anti-TNF (n = 45)	Anti-IL-17 (n = 43)	Anti-IL-23 (n = 27)	*p*-Value
GLY				
Baseline	6 (14.3), n = 42	13 (34.2), n = 38	12 (44.4), n = 27	0.0299
36 weeks	8 (21.6), n = 37	12 (32.4), n = 37	7 (30.4), n = 23	0.5945
60 weeks	5 (19.2), n = 26	9 (29), n = 31	4 (23.5), n = 17	0.7396
Dyslipidemia				
Baseline	16 (50.0), n = 32	20 (57.1), n = 35	7 (41.2), n = 17	0.5833
36 weeks	9 (69.2), n = 13	22 (81.5), n = 27	9 (64.3), n = 14	0.3998
60 weeks	5 (50.0), n = 10	16 (69.6), n = 23	7 (58.3), n = 12	0.5644
Serum creatinine				
Baseline	0 (0.0), n = 45	3 (7.0), n = 43	4 (14.8), n = 27	n.a.
36 weeks	1 (2.4), n = 42	3 (7.5), n = 40	3 (12.5), n = 24	0.2506
60 weeks	2 (6.5), n = 31	4 (12.1), n = 33	2 (10.5), n = 19	0.6806
Serum urea				
Baseline	0 (0.0), n = 29	8 (19.0), n = 42	3 (12.5), n = 24	n.a.
36 weeks	2 (6.5), n = 31	6 (15.0), n = 40	2 (8.3), n = 24	0.6288
60 weeks	1 (4.5), n = 22	6 (18.2), n = 33	3 (15.8), n = 19	0.2390

GLY—serum glucose level. Data are reported as the number of patients with elevated biomarker levels (%). Reported n corresponds to patients with complete data for the respective variables; n.a.—not applicable.

## Data Availability

The dataset analyzed in the current study is part of an ongoing Ph.D. study and is available from the corresponding author upon reasonable request.

## Abbreviations

The following abbreviations are used in this manuscript:

PASI	Psoriasis Area Severity Index
DLQI	The Dermatology Life Quality Index
BSA	Body Surface Area
PSSI	Psoriasis Scalp Severity Index
NAPSI	Nail Psoriasis Severity Index
GPSS	Genital Psoriasis Symptoms Scale
TNFα-IL23-Th17 axis	Tumor Necrosis Factor alpha -Interleukin-23 (IL-23)-T helper 17 (Th17) cells axis
anti-TNF	Anti-Tumor Necrosis Factor alpha biological therapy
anti-IL-17	Anti-Interleukin 17 biological therapy
anti-IL-23	Anti-Interleukin 23 biological therapy
anti-IL-12/23	Anti- interleukin 12/23 biological therapy
BMI	Body Mass Index
IMT	Intima-media thickness
MetS	Metabolic syndrome
PDE4i	Phosphodiesterase-4 inhibitor
MTX	Methotrexate

## References

[1] Boehncke W.H., Schönm M.P. (2015). Psoriasis. Lancet.

[2] Skayem C., Taieb C., Halioua B., Baissac C., Saint Aroman M. (2025). Epidemiology of Psoriasis: A Worldwide Global Study. Acta Derm. Venereol..

[3] Parisi R., Iskandar I.Y.K., Kontopantelis E., Augustin M., Griffiths C.E.M., Ashcroft D.M., Global Psoriasis Atlas (2020). National, regional, and worldwide epidemiology of psoriasis: Systematic analysis and modelling study. BMJ.

[4] Bartosińska J., Szepietowski J.C., Raczkiewicz D., Griffiths C.E.M., Ashcroft D.M., Wright A.K., Podwójcic K., Turcza J., Maluchnik M., Chłoń-Domińczak A. (2025). Epidemiology of Psoriasis in Poland: Prevalence, Incidence, and Mortality Rates. Int. J. Dermatol..

[5] Nicolescu A.C., Bucur Ș., Giurcăneanu C., Gheucă-Solovăstru L., Constantin T., Furtunescu F., Ancuța I., Constantin M.M. (2021). Prevalence and Characteristics of Psoriasis in Romania—First Study in Overall Population. J. Pers. Med..

[6] Boca A.N., Ilies R.F., Vesa S., Pop R., Tataru A.D., Buzoianu A.D. (2019). The first nation-wide study revealing epidemiologic data and life quality aspects of psoriasis in Romania. Exp. Ther. Med..

[7] Parisi R., Symmons D.P., Griffiths C.E., Ashcroft D.M., IMPACT Project Team (2013). Global epidemiology of psoriasis: A systematic review of incidence and prevalence. J. Investig. Dermatol..

[8] Sarac G., Koca T.T., Baglan T. (2016). A brief summary of clinical types of psoriasis. North Clin. Istanb..

[9] Kimmel G.W., Lebwohl M., Bhutani T., Liao W., Nakamura M. (2018). Psoriasis: Overview and Diagnosis. Evidence-Based Psoriasis.

[10] Wozel G., Klein E., Mrowietz U., Reich K., Sebastian M., Streit V. (2011). Scalp psoriasis. J. Dtsch. Dermatol. Ges..

[11] Rich P., Scher R.K. (2003). Nail Psoriasis Severity Index: A useful tool for evaluation of nail psoriasis. J. Am. Acad. Dermatol..

[12] Gottlieb A.B., Kirby B., Ryan C., Naegeli A.N., Burge R., Potts Bleakman A., Anatchkova M.D., Yosipovitch G. (2018). The Development of a Patient-Reported Outcome Measure for Assessment of Genital Psoriasis Symptoms: The Genital Psoriasis Symptoms Scale (GPSS). Dermatol. Ther..

[13] Liluashvili S., Kituashvili T. (2019). Dermatology Life Quality Index and disease coping strategies in psoriasis patients. Postep. Dermatol. Alergol..

[14] Nast A., Smith C., Spuls P.I., Avila Valle G., Bata-Csörgö Z., Boonen H., De Jong E., Garcia-Doval I., Gisondi P., Kaur-Knudsen D. (2020). EuroGuiDerm Guideline on the systemic treatment of Psoriasis vulgaris—Part 1. J. Eur. Acad. Dermatol. Venereol..

[15] Sugumaran D., Yong A.C.H., Stanslas J. (2024). Advances in psoriasis research: From pathogenesis to therapeutics. Life Sci..

[16] Armstrong A.W., Blauvelt A., Callis Duffin K., Huang H.Y., Savage J.L., Guo L., Merola J.F. (2025). Psoriasis. Nat. Rev. Dis. Primers.

[17] Dand N., Stuart P.E., Bowes J., Ellinghaus D., Nititham J., Saklatvala J.R., Teder-Laving M., Thomas L.F., Traks T., Uebe S. (2025). GWAS meta-analysis of psoriasis identifies new susceptibility alleles impacting disease mechanisms and therapeutic targets. Nat. Commun..

[18] Liu S., He M., Jiang J., Duan X., Chai B., Zhang J., Tao Q., Chen H. (2024). Triggers for the onset and recurrence of psoriasis: A review and update. Cell Commun. Signal..

[19] Rendon A., Schäkel K. (2019). Psoriasis Pathogenesis and Treatment. Int. J. Mol. Sci..

[20] Ten Bergen L.L., Petrovic A., Krogh Aarebrot A., Appel S. (2020). The TNF/IL-23/IL-17 axis—Head-to-head trials comparing different biologics in psoriasis treatment. Scand. J. Immunol..

[21] Brownstone N.D., Hong J., Mosca M., Hadeler E., Liao W., Bhutani T., Koo J. (2021). Biologic Treatments of Psoriasis: An Update for the Clinician. Biologics.

[22] Floris A., Mugheddu C., Sichi L., Anedda J., Frau A., Sorgia J., Li Volsi L., Paladino M.T., Congia M., Chessa E. (2025). Treatment of psoriasis with different classes of biologics reduces the likelihood of peripheral and axial psoriatic arthritis development. Rheumatology.

[23] Amara S., Pasumarthi A., Parikh N., Kodali N., Lebwohl M., Monks G. (2025). Psoriasis management tree based on comorbidity. Int. J. Dermatol..

[24] Osigwe P.C., Agomoh C.E., Osigwe I.S., Akumiah F.K. (2024). The Association Between Psoriasis and Atherosclerotic Cardiovascular Disease: A Systematic Review and Meta-Analysis of Observational Studies. Cureus.

[25] Dattola A., Manenti G., Ferrari D., Vollono L., Marsico S., Lamacchia F., Esposito M., Marchesano M., Zangrilli A., Floris R. (2022). Prevalence of Atherosclerosis in Psoriatic Patients Detected with Epiaortic Color Doppler Ultrasound and Computed Tomography Angiography. Dermatol. Pract. Concept..

[26] Liu C., Chen H., Liu Y., Huang H., Yu W., Du T., Long X., Chen X., Chen Z., Guo S. (2022). Immunity: Psoriasis comorbid with atherosclerosis. Front. Immunol..

[27] Su W., Zhao Y., Wei Y., Zhang X., Ji J., Yang S. (2021). Exploring the Pathogenesis of Psoriasis Complicated with Atherosclerosis via Microarray Data Analysis. Front. Immunol..

[28] Dascălu R.C., Bărbulescu A.L., Dinescu Ș.C., Biță C.E., Stoica L.E., Sandu R.E., Vreju F.A. (2024). Metabolic Syndrome in Psoriasis Patients—An Observational Study. Curr. Health Sci. J..

[29] Bucur S., Serban E.D., Ileanu B.V., Costache R.S., Nicolescu A.C., Constantin T., Costache D.O., Constantin M.M. (2024). Effectiveness and Drug Survival of Ixekizumab and Secukinumab in Patients with Moderate to Severe Plaque Psoriasis. Psoriasis.

[30] Mihu C., Popescu C.A., Neag M.A., Bocșan I.C., Melincovici C.S., Baican A.L., Năsui B.A., Buzoianu A.D. (2023). The Psoriasis Disability Index in Romanian Psoriasis Patients during COVID-19 Pandemic. J. Clin. Med..

[31] Decean L., Badea M., Ilies R., Sasu A., Rus V., Mihai A. (2022). Psoriasis-Related Stigma: Is There More to Uncover?. J. Interdiscip. Med..

[32] Burlacu G., Virag-Iorga C., Radu M.O., Giurcaneanu C. (2025). Strategies to improve quality of life in psoriasis. DermatoVenerologia.

[33] COD PROTOCOL DENUMIRE [Internet]. [Cited 18 January 2026]. https://cnas.ro/wp-content/uploads/2025/06/lista-protocoalelor-terapeutice-iunie-2025-site_all.pdf.

[34] Raharja A., Mahil S.K., Barker J.N. (2021). Psoriasis: A brief overview. Clin. Med..

[35] Daugaard C., Iversen L., Hjuler K.F. (2022). Comorbidity in Adult Psoriasis: Considerations for the Clinician. Psoriasis.

[36] Cai J., Cui L., Wang Y., Li Y., Zhang X., Shi Y. (2021). Cardiometabolic Comorbidities in Patients with Psoriasis. Front. Pharmacol..

[37] de Ruiter C.C., Rustemeyer T. (2022). Biologics Can Significantly Improve Dermatology Life Quality Index in Psoriatic Patients. Psoriasis.

[38] Timis T.L., Beni L., Mocan T., Florian I.A., Orasan R.I. (2023). Biologic Therapies Decrease Disease Severity and Improve Depression and Anxiety Symptoms in Psoriasis Patients. Life.

[39] Wang H., Hou S., Kang X., Yu C., Yang B., Shi Y., Li F., Li W., Gu J., Lei M. (2025). BMI matters: Understanding the link between weight and severe psoriasis. Sci. Rep..

[40] Li L., Liu K., Duan X., Xu L., Yang Q., Liu F. (2023). A Comparison of Clinical Characteristics in Overweight/Obese and Normal Weight Patients with Psoriasis Vulgaris. Clin. Cosmet. Investig. Dermatol..

[41] Bardazzi F., Balestri R., Baldi E., Antonucci A., De Tommaso S., Patrizi A. (2010). Correlation between BMI and PASI in patients with moderate to severe psoriasis undergoing biological therapy. Dermatol. Ther..

[42] Haberman R.H., Ogdie A., Merola J.F., Scher J.U., Eder L. (2025). The Obesity-Inflammation Axis in Psoriatic Disease: Mechanisms and Therapeutic Strategies. Nat. Rev. Rheumatol..

[43] Almenara-Blasco M., Gracia-Cazaña T., Poblador-Plou B., Laguna-Berna C., Carmona-Pírez J., Navarro-Bielsa A., Prados-Torres A., Gimeno-Miguel A., Gilaberte Y. (2024). Multimorbidity of Psoriasis: A Large-Scale Population Study of Its Associated Comorbidities. J. Clin. Med..

[44] Bruner C.R., Feldman S.R., Ventrapragada M., Fleischer A.B. (2003). A systematic review of adverse effects associated with topical treatments for psoriasis. Dermatol. Online J..

[45] West J., Ogston S., Foerster J. (2016). Safety and Efficacy of Methotrexate in Psoriasis: A Meta-Analysis of Published Trials. PLoS ONE.

[46] Armstrong A.W., Patel M., Li C., Garg V., Mandava M.R., Wu J.J. (2023). Real-world switching patterns and associated characteristics in patients with psoriasis treated with biologics in the United States. J. Dermatolog. Treat..

[47] Mease P.J., Blauvelt A., Sima A.P., Beaty S.W., Low R., Gomez B., Gurrola M., Lebwohl M.G. (2024). Impact of Disease Factors of Patients with Psoriasis and Psoriatic Arthritis on Biologic Therapy Switching. Dermatol. Ther..

[48] Graier T., Salmhofer W., Jonak C., Weger W., Zikeli C., Gruber B., Sator P., Prillinger K., Mlynek A., Schütz-Bergmayr M. (2023). Evolution of characteristics and biologic treatment effectiveness in patients of the Austrian psoriasis registry from 2004–2022. J. Dtsch. Dermatol. Ges..

[49] Ntawuyamara E., Deng B., Liang Y. (2025). Cutaneous and systemic improvements in psoriasis patients after different biologic treatments. Sci. Rep..

[50] Blauvelt A., Gondo G.C., Bell S., Echeverría C., Schmitt-Egenolf M., Skov L., van de Kerkhof P., McCormick Howard L., Strober B. (2023). Psoriasis Involving Special Areas Is Associated with Worse Quality of Life. J. Psoriasis Psoriatic Arthritis.

[51] Khan M., Wallace C.E., Ahmed F., Rahman S.M., Memon N., Haque A. (2024). Assessing Comparative Efficacy of Biologics for the Treatment of Psoriasis with Nail Involvement. J. Psoriasis Psoriatic Arthritis.

[52] Papadimitriou I., Bakirtzi K., Katoulis A., Ioannides D. (2021). Scalp Psoriasis and Biologic Agents: A Review. Skin Appendage Disord..

[53] Ordin 1218/16.09.2010. Portal Legislativ. https://legislatie.just.ro/public/DetaliiDocument/123112.

[54] Eli Lilly România Anunță Lansarea TALTZ în Tratamentul Psoriazisului Moderat-Sever. MedicalManager 2018. https://www.medicalmanager.ro/eli-lilly-romania-anunta-lansarea-taltz-in-tratamentul-psoriazis-ului-moderat-sever/.

[55] Anexa 28/10/2022. Portal Legislativ. https://legislatie.just.ro/Public/DetaliiDocumentAfis/261213.

[56] Casa Națională de Asigurări de Sănătate (CNAS) Protocol List. https://cnas.ro/wp-content/uploads/2022/04/Binder1-3.pdf.

